# Supplementary material for: Peptide density targets and impedes triple negative breast cancer metastasis
Source: Nat Commun. 2018 Jul 4;9:2612. doi: 10.1038/s41467-018-05035-5 (PMC6031661; doi:10.1038/s41467-018-05035-5)
Supplement: Supplementary file 1 — Supplementary Information [file 41467_2018_5035_MOESM1_ESM.pdf]

Supplementary Information

Peptide density targets and impedes triple negative breast cancer metastasis

Daxing Liu[1, 2], Peng Guo[1, 3, 4], Craig McCarthy[1],

Biran Wang[1], Yu Tao [1], Debra Auguste[1, 2, *]

1 Department of Biomedical Engineering, the City College of New York. New York, New York 10031, United States.

2 Current: Department of Chemical Engineering, Northeastern University. 360 Huntington Avenue, Boston, MA 02115

3 Vascular Biology Program, Boston Children's Hospital, 300 Longwood Avenue, Boston, MA 02115, United States

4 Department of Surgery, Harvard Medical School and Boston Children's Hospital, 300 Longwood Avenue, Boston, MA 02115, United States

*Corresponding author: Debra Auguste, 225 ISEC, Department of Chemical Engineering, Northeastern University. Phone: +1-617-373-6243, email: d.auguste@northeastern.edu

Contents

Supplementary Figure 1 Characterization of the DV1-N_3 peptide

Supplementary Table 1 Breast cancer cell surface CXCR4 density

Supplementary Figure 2 Cytotoxicity evaluation of DV1-N_3 and sDV-N_3

Supplementary Figure 3 Synthesis and NMR characterization of lipid-PEG-DBCO

Supplementary Figure 4 Surface morphology of liposomes

Supplementary Table 2 Characterization of DV1-functionalized liposomes

Supplementary Figure 5 AFM adhesion force distribution maps and force-displacement curves

Supplementary Figure 6 Binding of L-DBCO and L-DV1-24k to breast cells using flow cytometry

Supplementary Figure 7 Ex vivo targeting effect of L-DV1 and CXCR4 mAb on human patient samples

Supplementary Figure 8 Mechanism of L-DV1-24k for prevention of metastasis of MDA-MB-231-Luc

Supplementary Figure 9 *In vivo* suppression of MDA-MB-231-Luc metastasis by L-DBCO, DV1-N_3, and LY2510924

Supplementary Figure 10 Tumour metastasis organ analysis

Supplementary Figure 11 Ex vivo analysis of lung metastasis

Supplementary Figure 12 Tumour growth trend of the breast orthotopic implantation model

Supplementary Figure 13 Cardiac pathology by H&E and TUNEL staining

Supplementary Figure 14 $CD34^+$ cell migration and CXCR4 expression after DV1-N_3 and LY2510924 administration

(a)

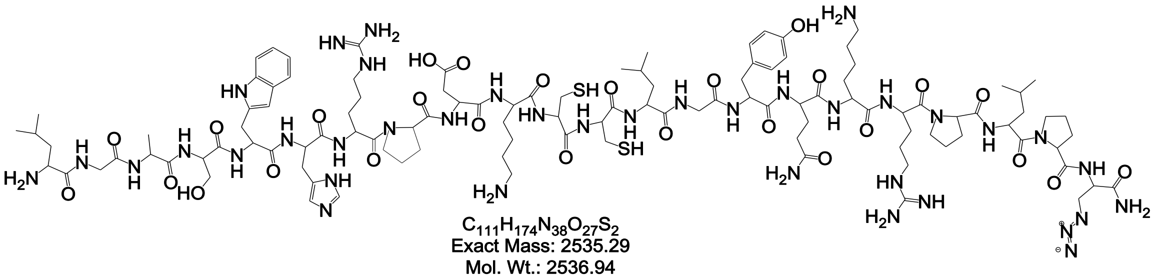
C111H174N38O27S2
Exact Mass: 2535.29
Mol. Wt.: 2536.94

(b)

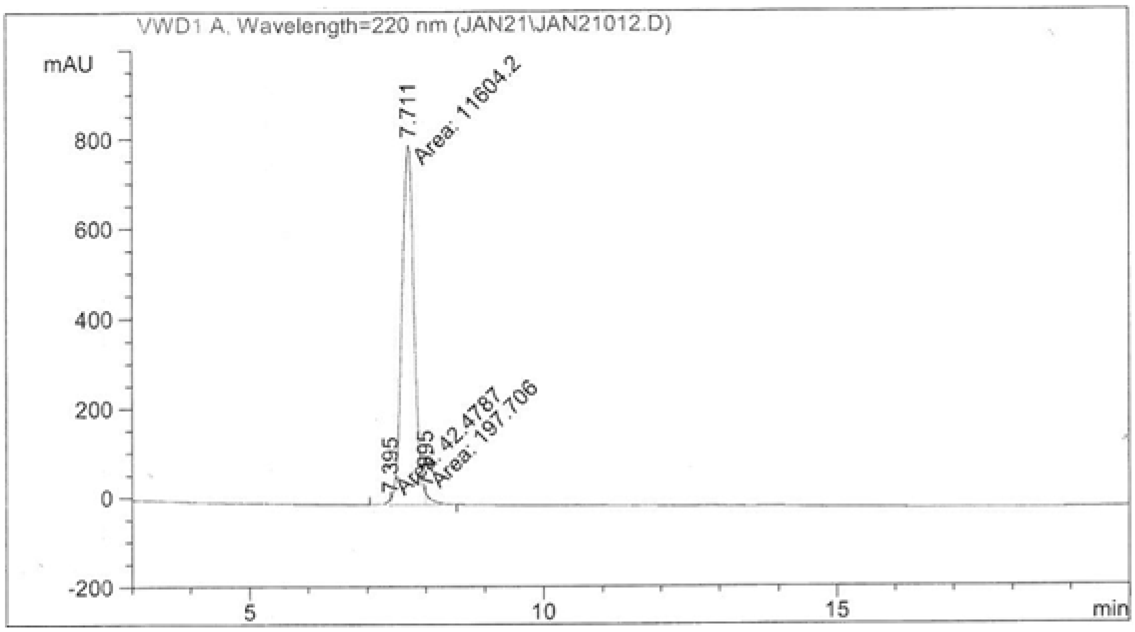
VWD1 A, Wavelength=220 nm (JAN21\JAN21012.D)
mAU
800
600
400
200
0
-200
5
10
15
min
7.711
Area: 11604.2
7.395
Area: 42.4787
Area: 197.706

(c)

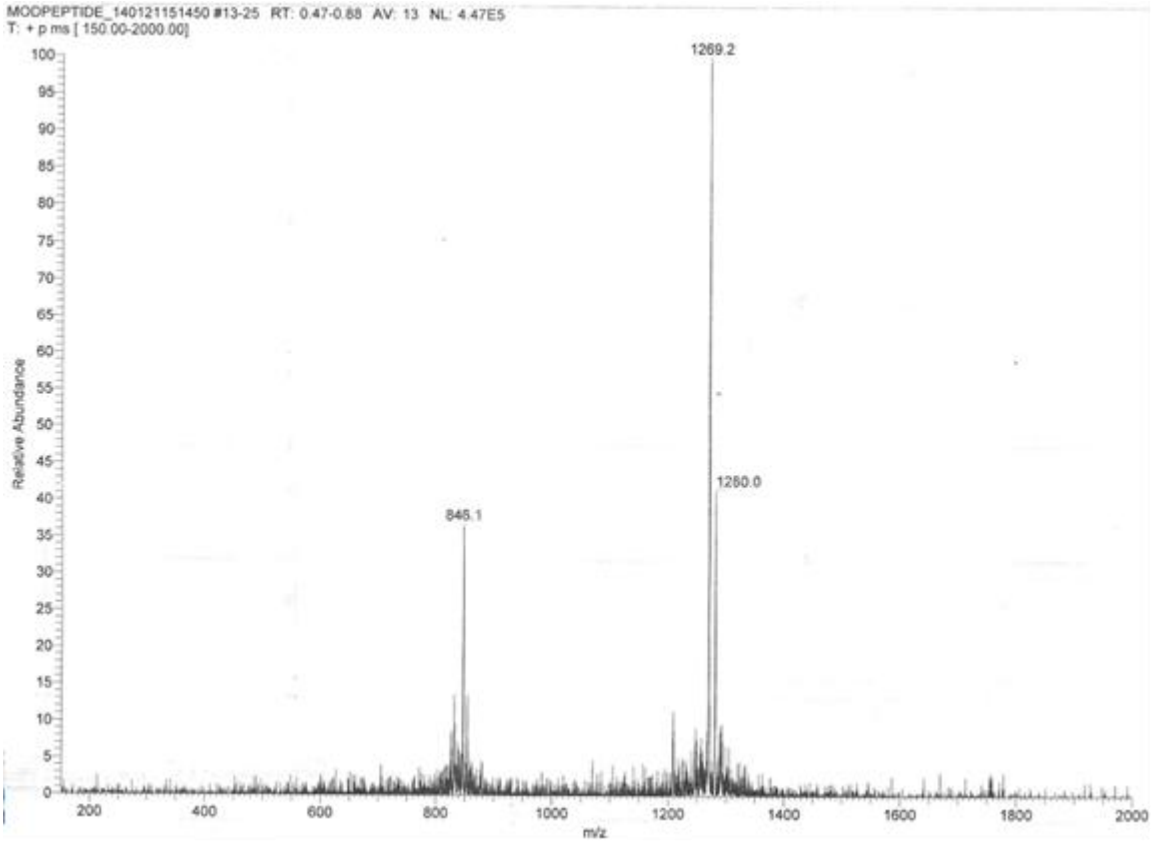

(d)

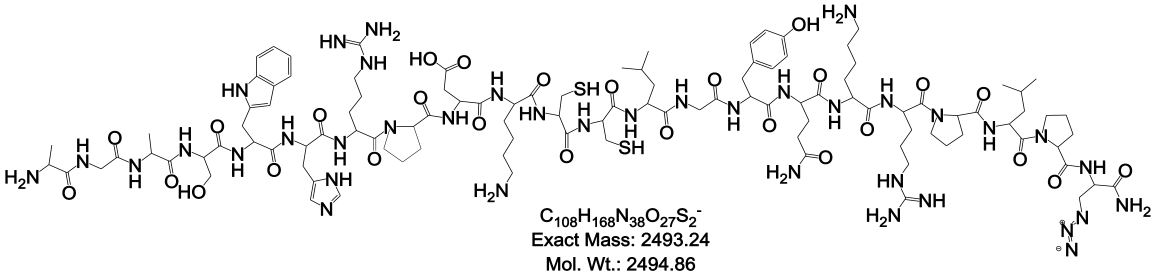

Supplementary Figure 1 **DV1-N3 and scrambled DV1-N3 peptides. a,** molecular structure of DV1-N_3 peptide (sequence: *L*-G-*A*-*S*-*W*-*H*-*R*-*P*-*D*-*K*-*C*-*C*-*L*-G-*Y*-*Q*-*K*-*R*-*P*-*L*-*P*-A (β-azido)-$CONH_2$ (all D-amino acids except G and the A (β-azido)), **b,** high performance liquid chromatography of DV1-N_3. **c,** mass spectrometry DV1-N_3 peptide, **d,** molecular structure of scrambled DV1-N_3 peptide (sequence: *A*-G-*A*-*S*-*W*-*H*-*R*-*P*-*D*-*K*-*C*-*C*-*L*-G-*Y*-*Q*-*K*-*R*-*P*-*L*-*P*-A (β-azido)-$CONH_2$.

Supplementary Table 1 Breast cancer cell surface CXCR4 density.

Cell line	HER 2	ER	PR	CXCR4 density (molecules/cell)
MCF-10A	-	-	-	4600 ± 100
MDA-MB-231	-	-	-	15000 ± 1000
MDA-MB-436	-	-	-	59000 ± 1400

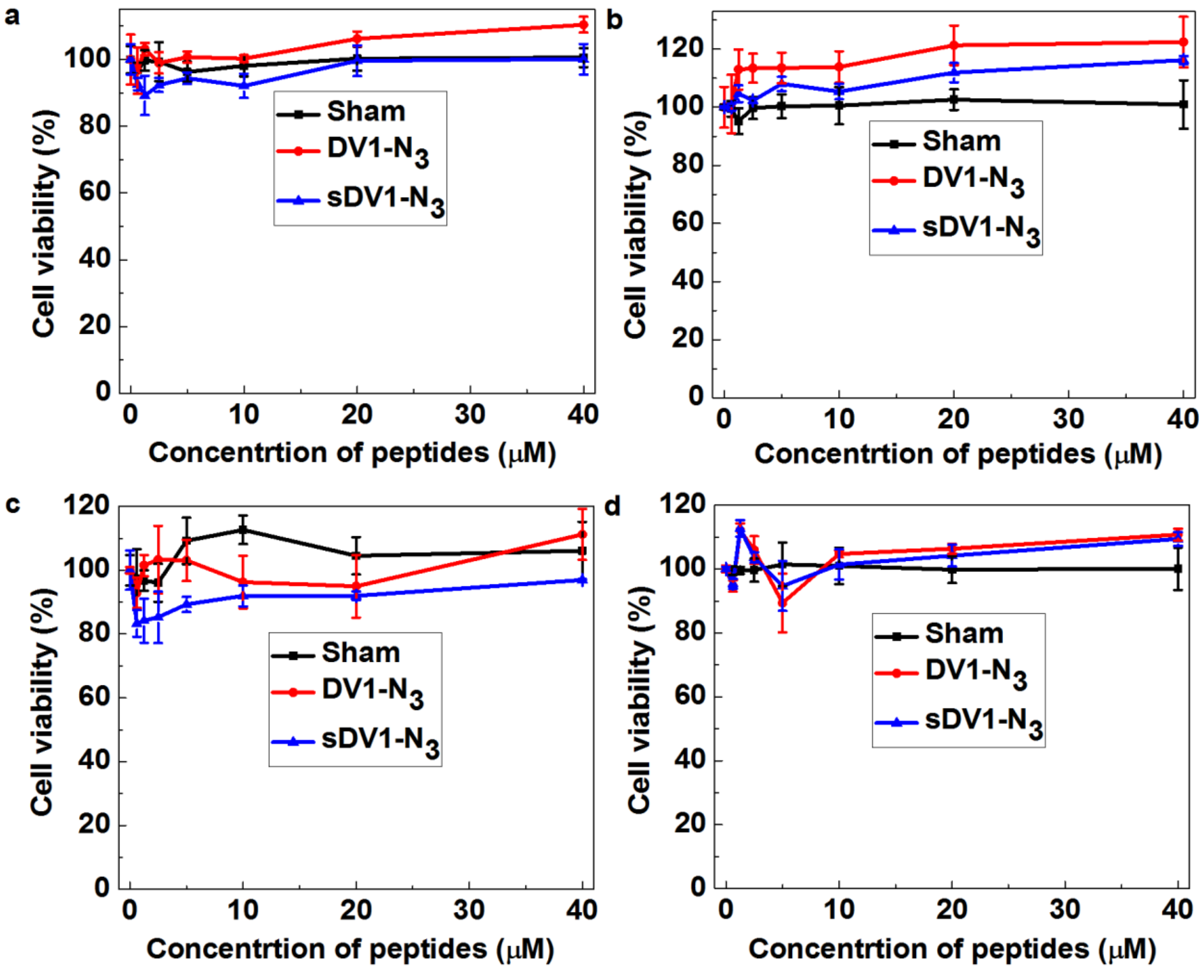

Supplementary Figure 2 **Cytotoxicity of DV1-N_3 and sDV1-N_3 peptides.** Cell viability of DV1-N_3 and sDV1-N_3 on (a) MDA-MB-231 at 24h; (b) MDA-MB-231 at 48h; (c) HUVECs at 24h; (d) HUVECs at 48h.

(a)

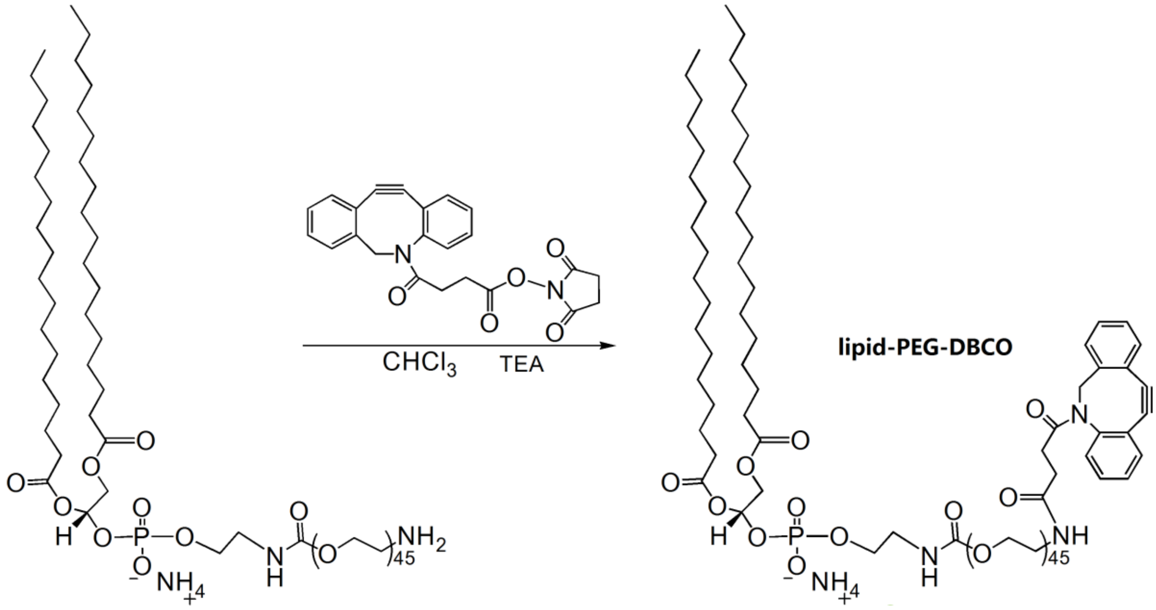
CHCl3
TEA
lipid-PEG-DBCO

(b)

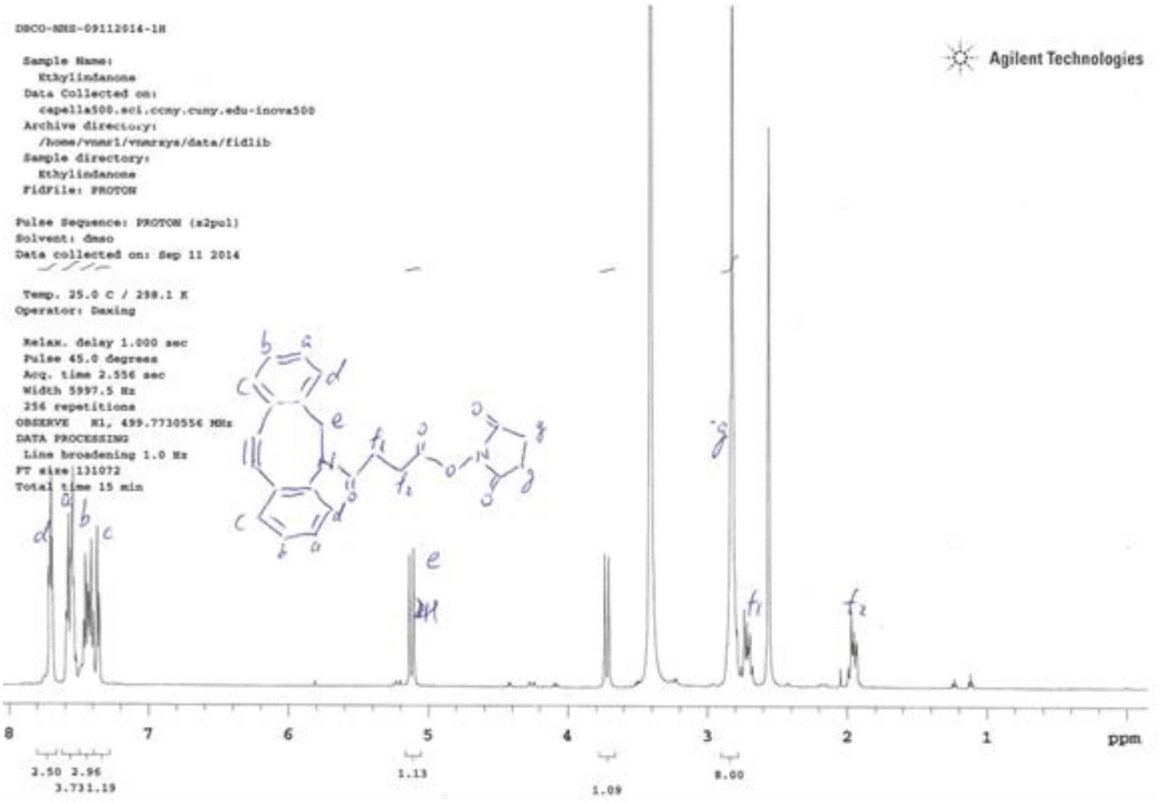
Agilent Technologies
Sample Name:
Ethylindanone
Pulse Sequence: PROTON (s2pul)
Solvent: dmso
Data collected on: Sep 11 2014
ppm

(c)

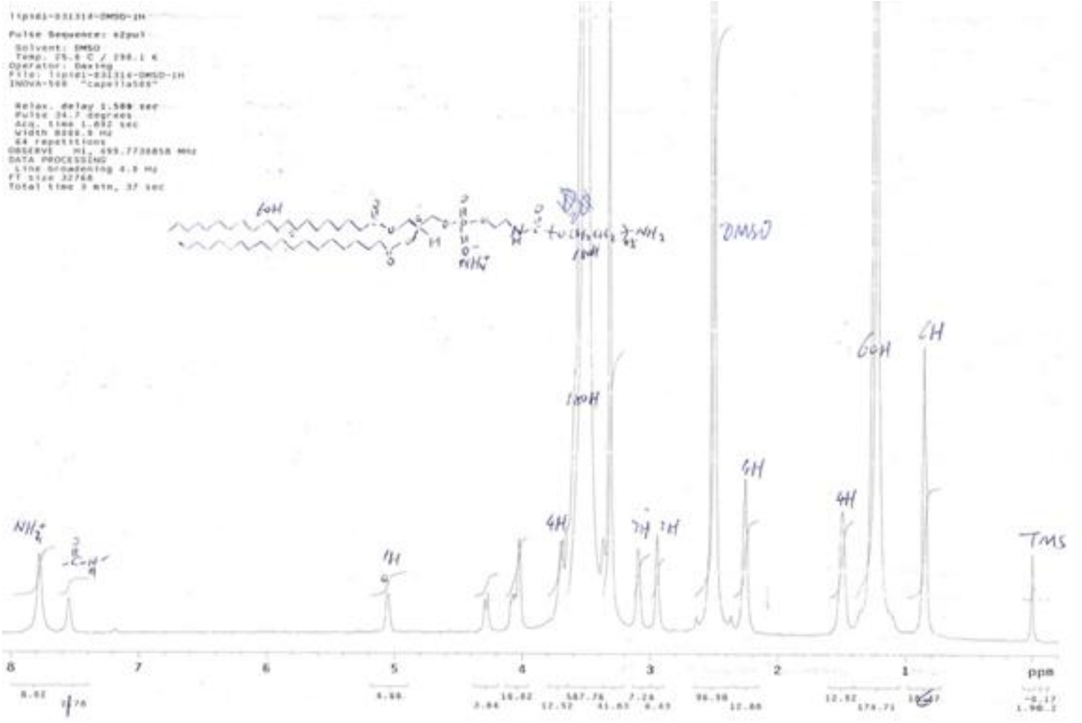

(d)

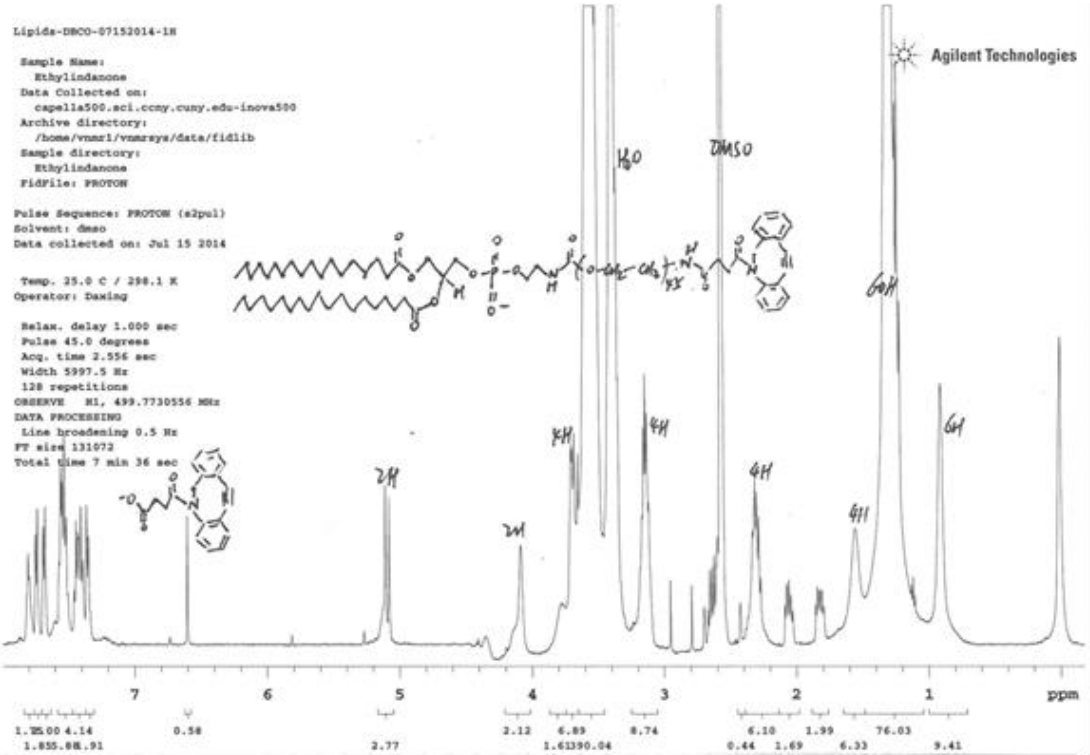

Supplementary Figure 3 **DSPE-PEG$_{2000}$-DBCO synthesis and 1H NMR characterization. a,** The synthesized procedure of DSPE-PEG$_{2000}$-DBCO. **b,** NMR spectrum of DBCO-NHS. c, NMR spectrum of DSPE-PEG$_{2000}$-NH$_2$. d, NMR spectrum of DSPE-PEG$_{2000}$-DBCO.

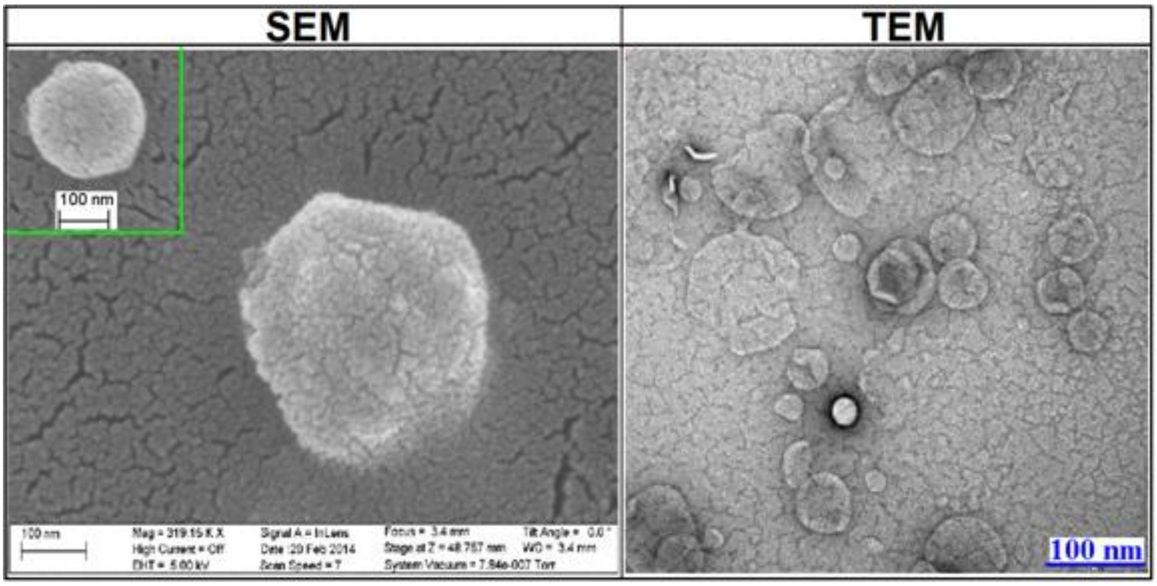

Supplementary Figure 4 **Characterization of liposome diameter and unilamellarity. a,** The morphology of the liposome by scanning electron microscopy (SEM), inset, diameter approximately 200 nm. **b,** Transmission electron microscopy (TEM) demonstrated unilamellar vesicles.

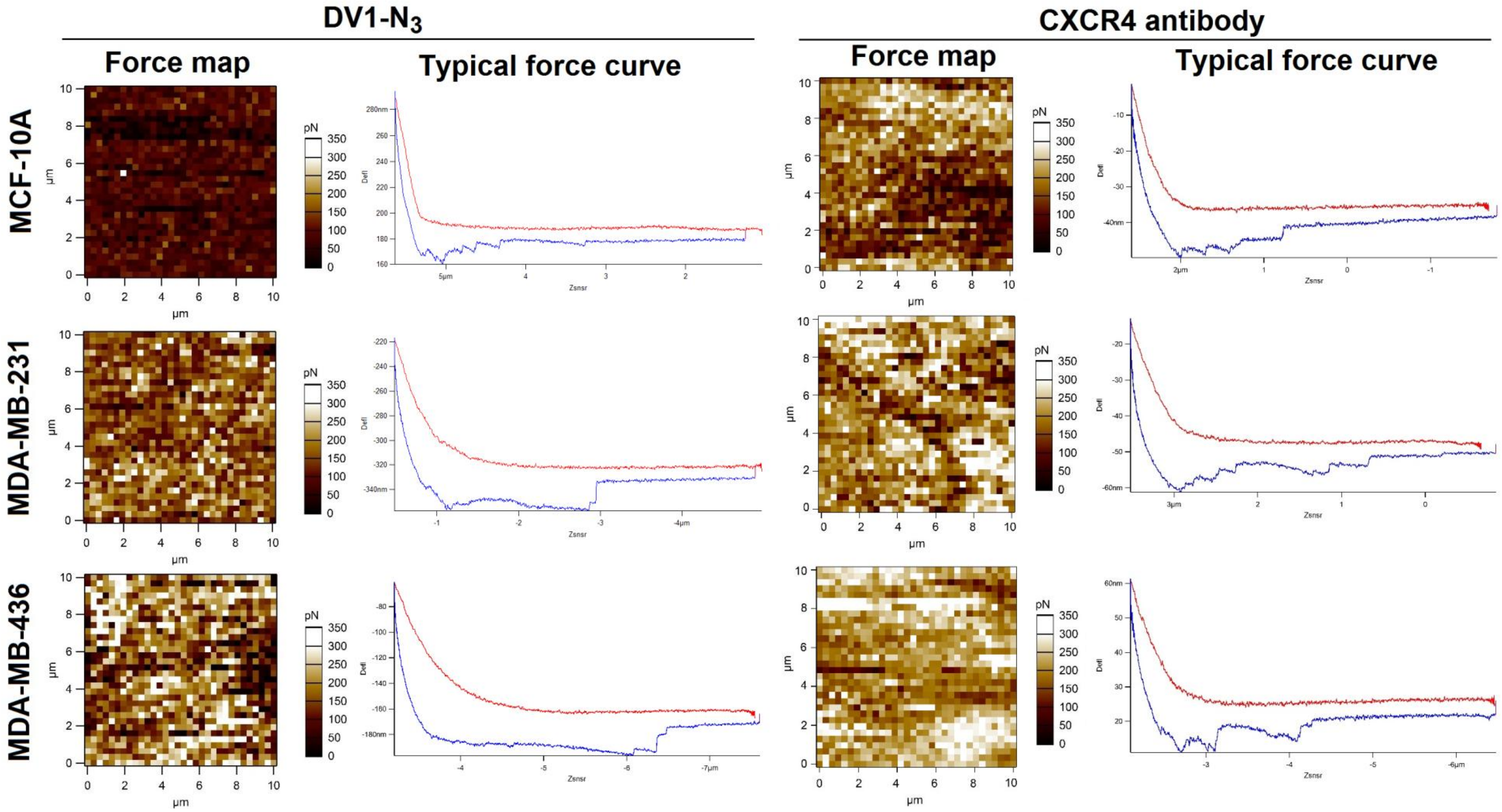

Supplementary Figure 5 AFM adhesion force distribution maps and force displacement curves. Atomic force microscopy between AFM tips modified with DV1 peptides or CXCR4 antibody and live MCF-10A, MDA-MB-231, and MDA-MB-436 cells were conducted.

Supplementary Table 2 Characterization of DV1-functionalized liposomes.

Liposome sample	Size (nm)	PDI	Zeta (mV)	DV1-N_3 concentration (μM)	Number of DV1-N_3 per liposome*	Number of DV1-N_3 per μm^2	Average distance per two peptides (Å)
L-DBCO	86.3 ± 1.7	0.313	-6.26 ± 1.10	0	0	0	-
L-DV1-9k	94.4 ± 0.6	0.243	-17.21 ± 1.26	11.2	262 ± 5	9×10^3	71
L-DV1-24k	99.8 ± 0.4	0.267	-13.99 ± 1.33	28.3	738 ± 6	24×10^3	45
L-DV1-39k	97.0 ± 0.4	0.240	-7.85 ± 1.28	47	1158 ±10	39×10^3	35
L-DV1-53k	98.6 ± 0.4	0.262	-6.03 ± 0.88	63.8	1624 ± 13	53×10^3	30
L-DV1-74k	100.1 ± 0.7	0.229	-7.95 ± 1.04	88.5	2322 ± 32	74×10^3	26

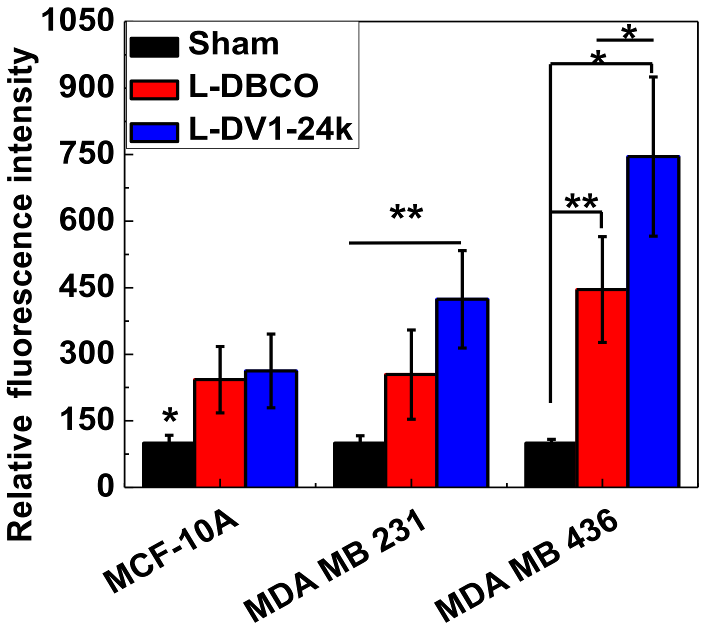

Supplementary Figure 6 **Binding of L-DBCO and L-DV1-24k to breast cells using flow cytometry.** Error bars represent ±1 s.d. of the mean. (n⩾3, *p<0.05, **p<0.01, by ANOVA with Tukey's post-test).

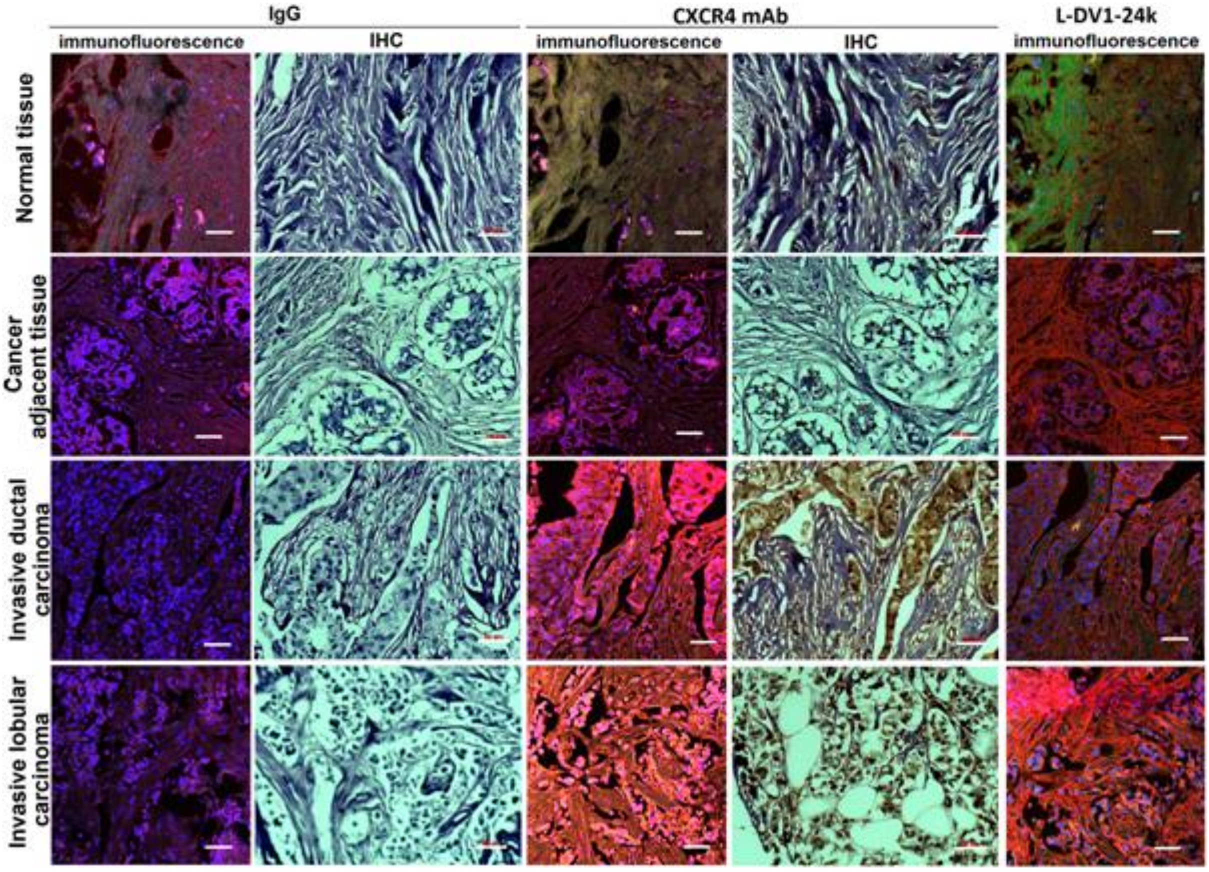

Supplementary Figure 7 **Ex vivo targeting effect of L-DV1-24k and CXCR4 mAb on human patient samples.** Immunohistology (IHC) and immunofluorescence staining of normal tissue, cancer adjacent tissue, invasive ductal carcinoma tissue and invasive lobular carcinoma tissue by IgG, CXCR4 mAb and L-DV1-24k (labeled with DiI). Scale bar=50 μm.

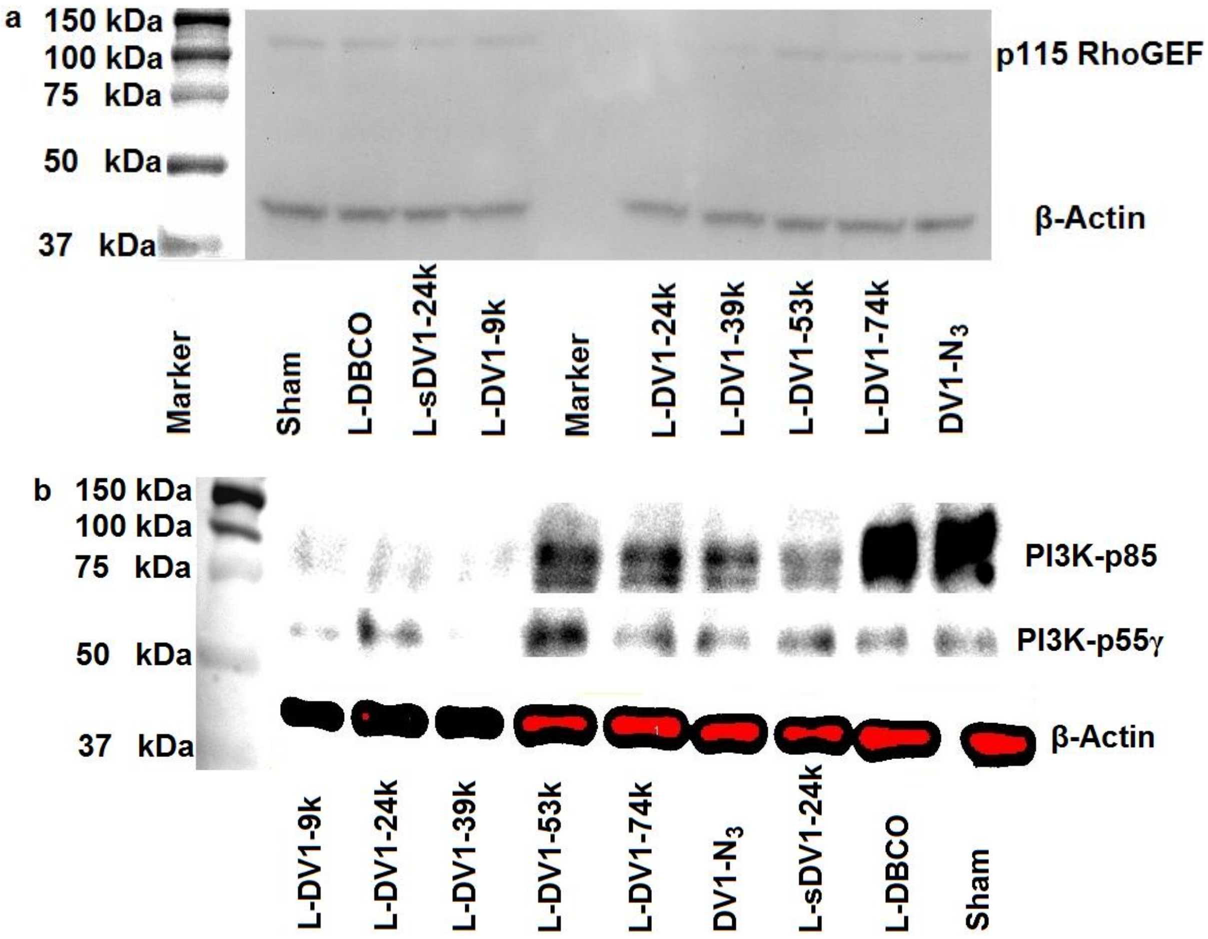

Supplementary Figure 8 **Western blot quantitation of the expression of p115-RhoGEF (a) and PI3K-p55γ and PI3K-p85 (b) relative to β-actin.**

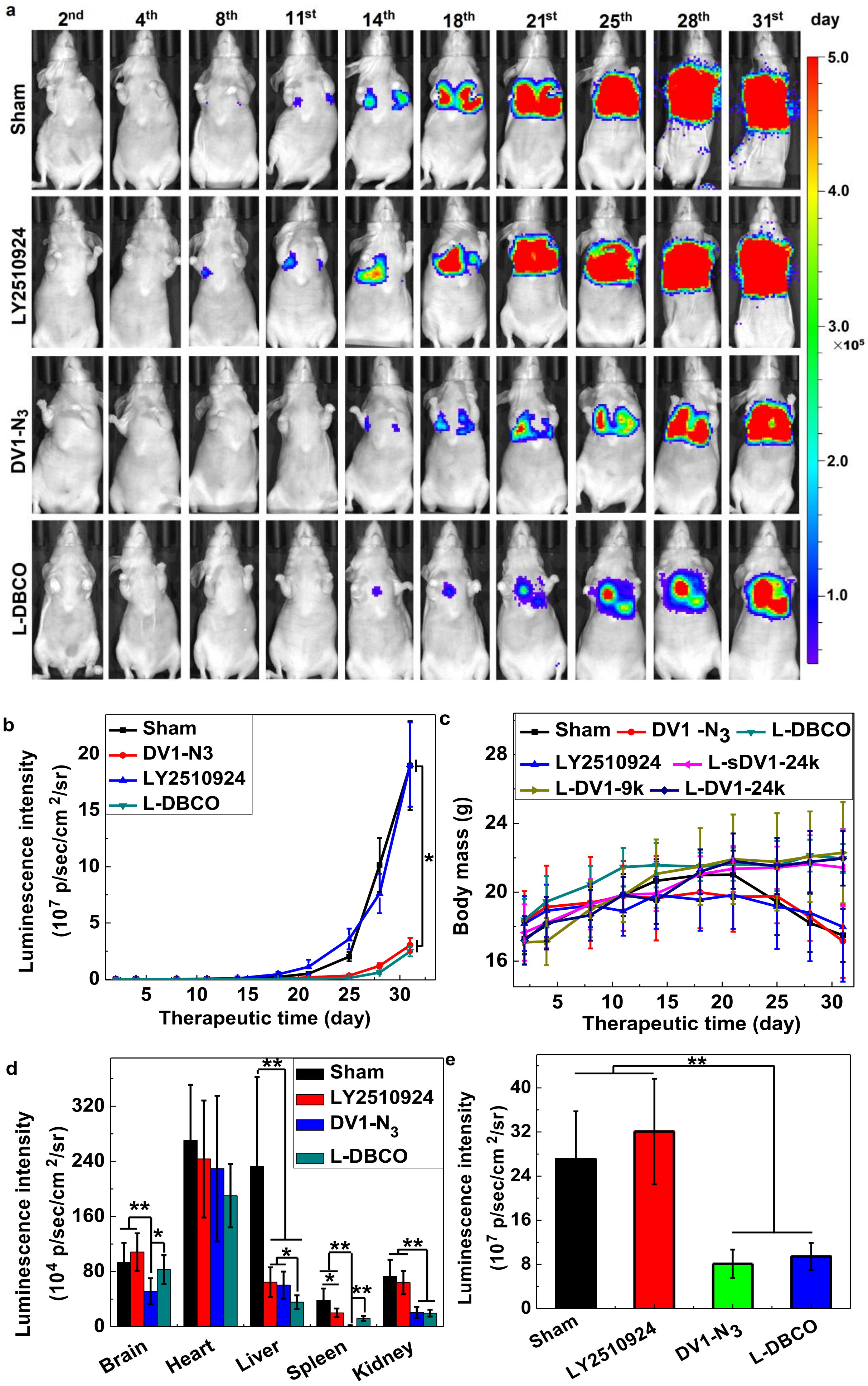
a
2nd 4th 8th 11st 14th 18th 21st 25th 28th 31st day
Sham
LY2510924
DV1-N3
L-DBCO
5.0
4.0
3.0
×10⁵
2.0
1.0
b
Sham
DV1-N3
LY2510924
L-DBCO
Luminescence intensity
(10⁷ p/sec/cm²/sr)
Therapeutic time (day)
c
Sham
DV1 -N3
L-DBCO
LY2510924
L-sDV1-24k
L-DV1-9k
L-DV1-24k
Body mass (g)
Therapeutic time (day)
d
Sham
LY2510924
DV1-N3
L-DBCO
Luminescence intensity
(10⁴ p/sec/cm²/sr)
Brain
Heart
Liver
Spleen
Kidney
e
Luminescence intensity
(10⁷ p/sec/cm²/sr)
Sham
LY2510924
DV1-N3
L-DBCO

Supplementary Figure 9 ***In vivo* suppression of MDA-MB-231-Luc metastasis by L-DBCO, DV1-N_3, and LY2510924.** MDA-MB-231-Luc cells treated with L-DBCO liposomes, DV1-N_3, and LY2510924 were injected via the tail vein to construct a metastatic breast cancer model Dosage: 6.7 mg/kg for DV1-N_3, and 3.3 mg/kg for LY2510924, equivalent to 30 μM, (the concentration equal to the concentration of DV1-N_3 peptides on L-DV1-24k). **a,** Tumor growth was quantified by luminescence. **b,** Quantitative characterization of lung metastasis growth. **c,** Body mass as a function time. **d,** Tumor metastasis signal at day 31 in excised brain, heart, liver, spleen and kidney. **e,** The lung metastasis signal at day 31. Error bars represent ±s.e.m. (n=6, *p<0.05, **p<0.01, by ANOVA with Tukey's post-test).

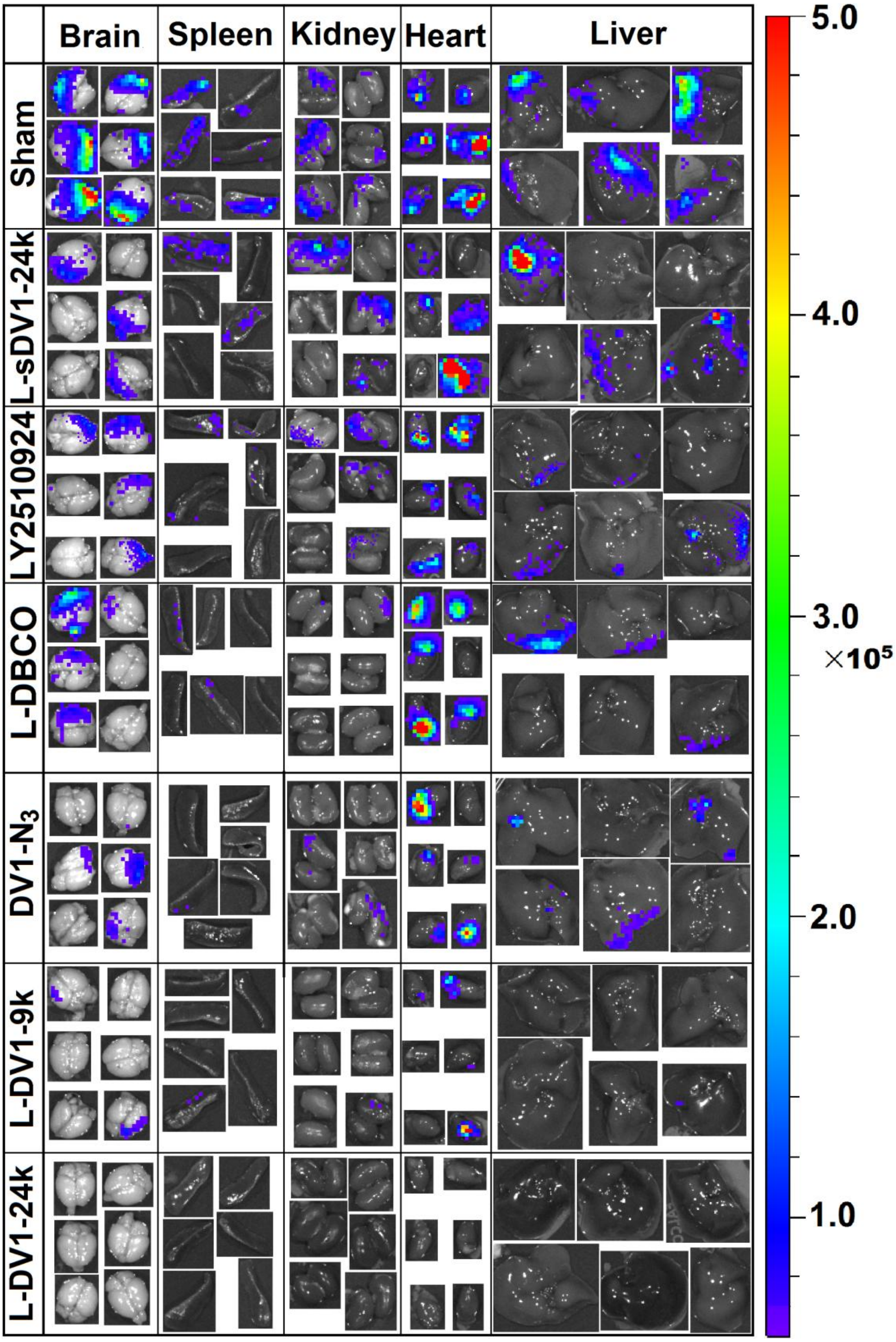

Supplementary Figure 10 **Tumour metastasis organ analysis**. Organs were excised from Sham, L-sDV-24k, LY2510924, DV1-N_3, L-DV1-9k and L-DV1-24k groups, and their luminescence of MDA-MB-231-Luc cells in brain, spleen, kidney, heart and liver were measured for metastatic characterization.

(a)

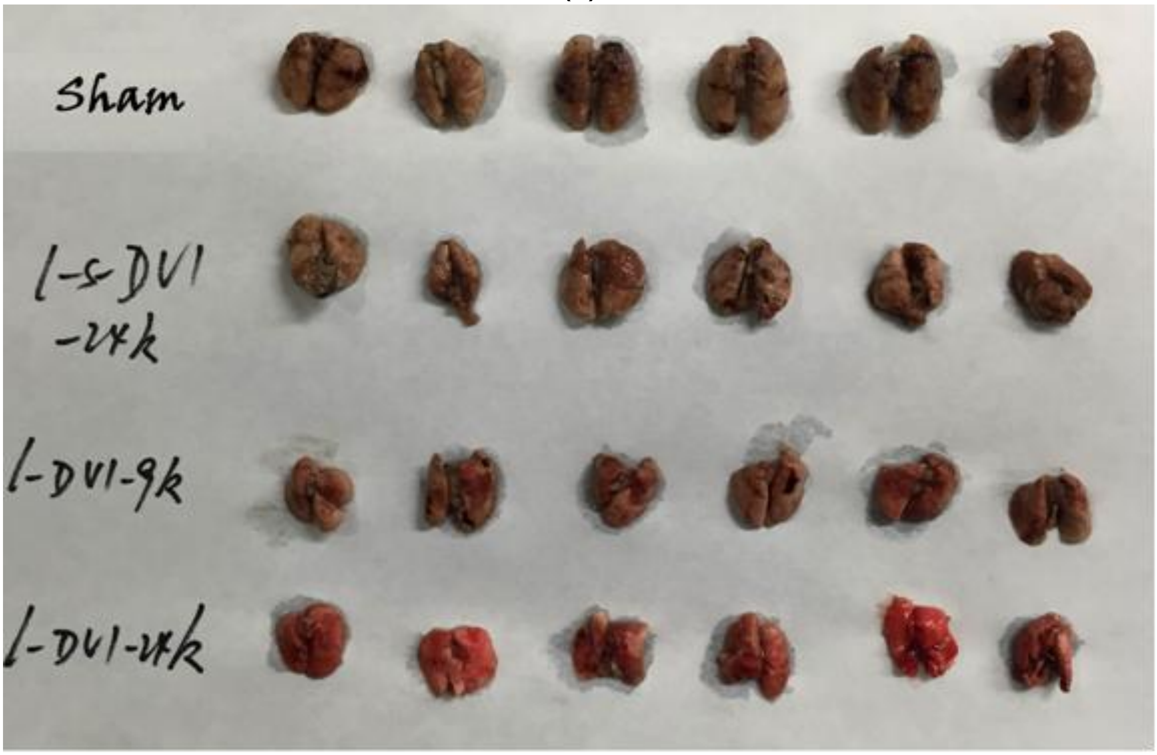

(b)

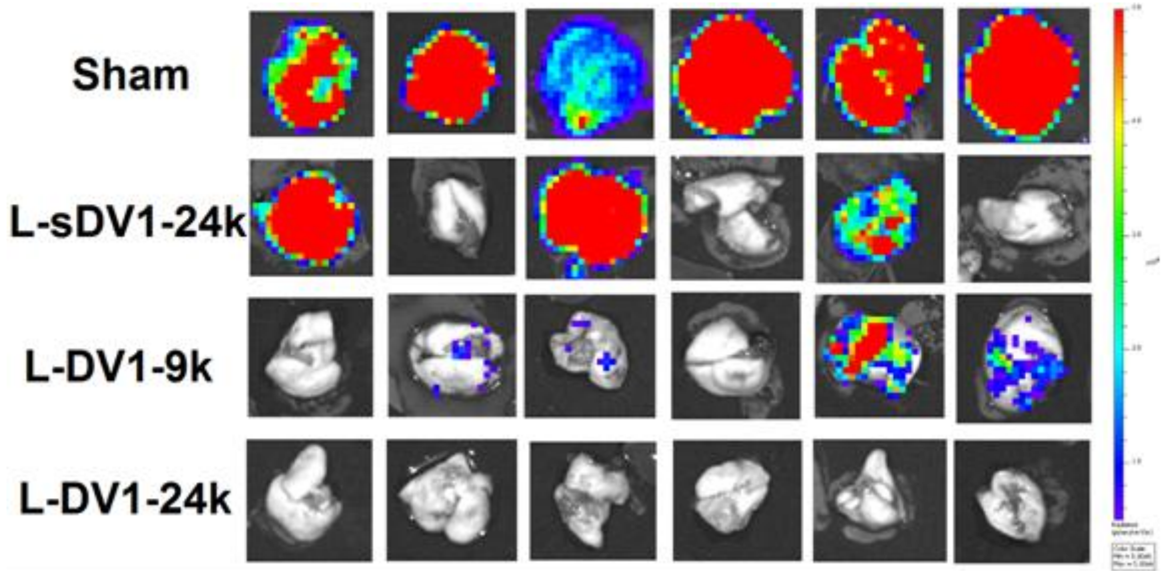

Supplementary Figure 11 ***Ex vivo* analysis of lung metastasis. a,** Lung size and appearance was evaluated post-mortem. **b,** Luminescence was measured in lungs excised from mice treated with sham, L-sDV1-24k, L-DV1-9k and L-DV1-24k groups at day 31.

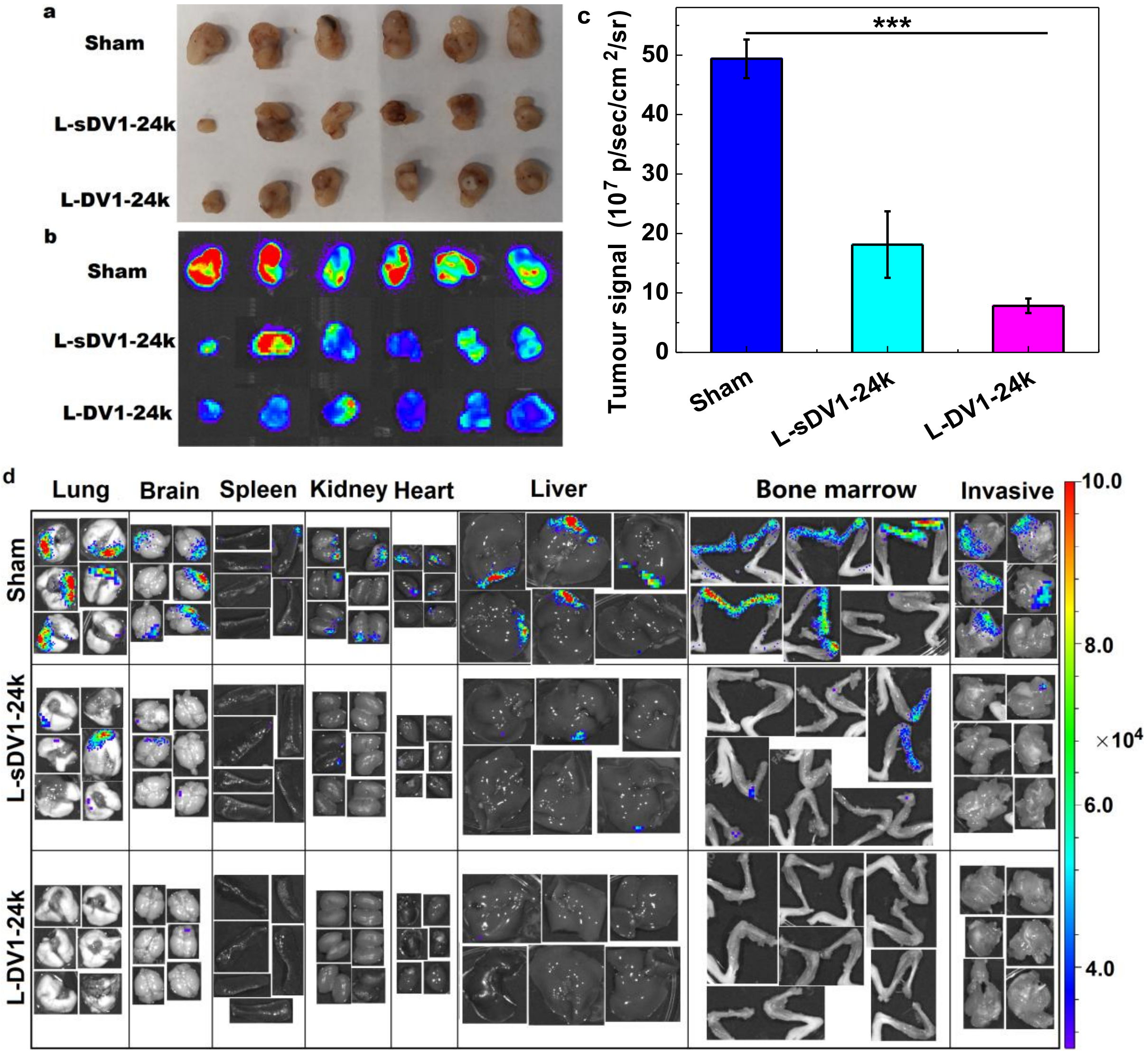

Supplementary Figure 12 ***In vivo* treatment of MDA-MB-231-Luc primary tumor by L-sDV1-24k and L-DV1-24k. a-b,** Visual comparison of the final tumour size and luminesence signal. **c,** Quantitative characterization of tumour signal intensity. **d,** Comparison of organ bioluminescence representative of MDA-MB-231-Luc metastasis to major organs and invasive to para-muscle. Error bars represent ± s.e.m. ($n \geq 6$, *$p<0.05$, **$p<0.01$, ***$p<0.001$ by ANOVA with Tukey's post-test).

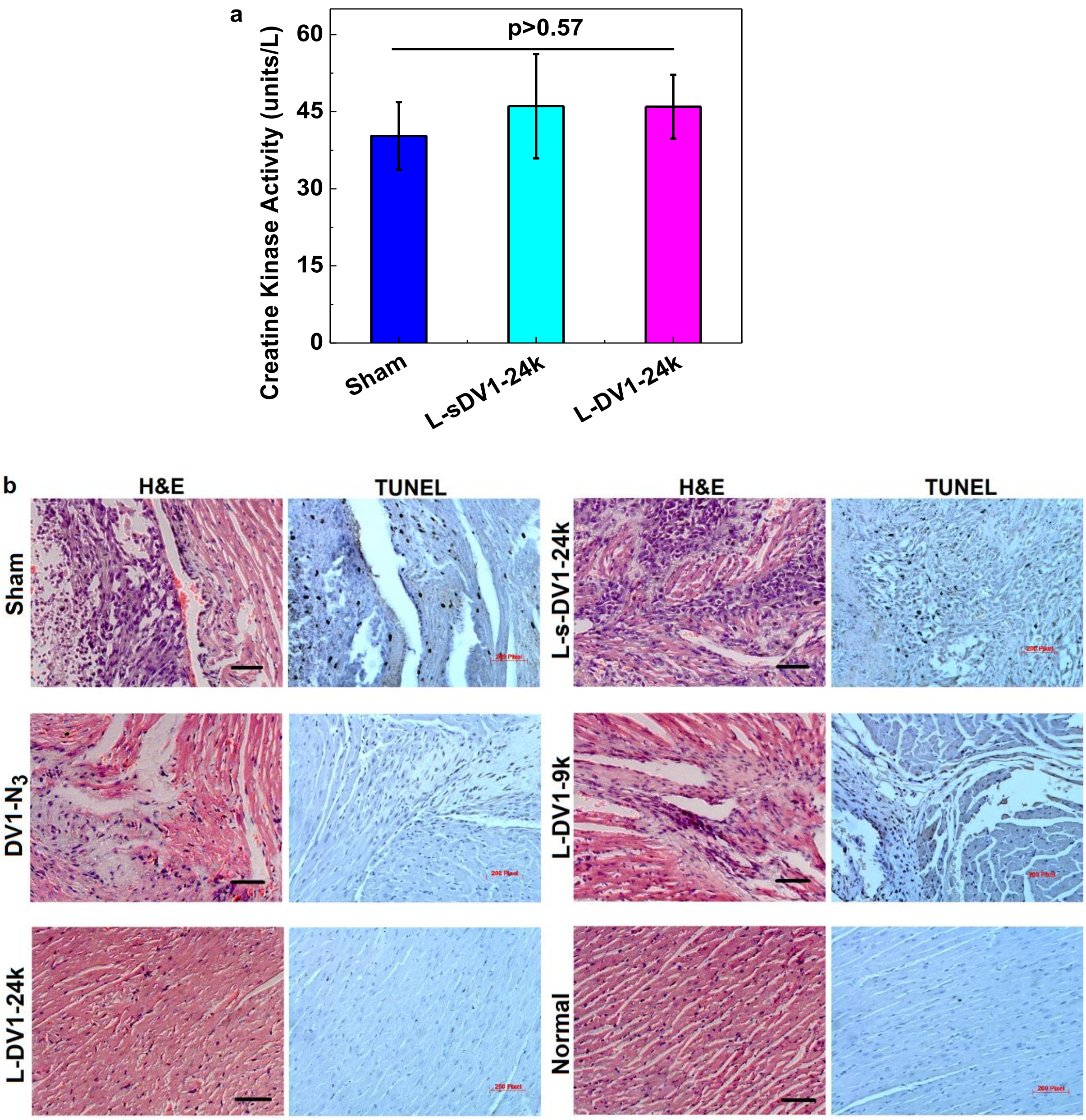

The positive control of TUNEL staining as below:

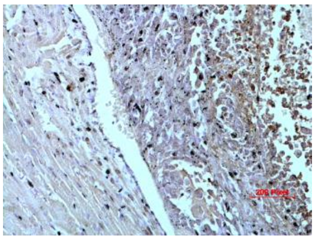

Supplementary Figure 13 **Cardiotoxicity test**. **a.** The serum creatine kinase activity test after the spontaneous metastasis prevention from a primary tumour study (completed after 24 days after 5 times of L-DV1 administration). **b.** Cardiac pathology by H&E and TUNEL staining after 31 days therapy for metastatic study. H&E and TUNEL double staining method to illustrate the heart morphology and the apoptosis rate induced by therapy of DV1-N_3, L-sDV1-24k, L-DV1-9k, L-DV1-24k.

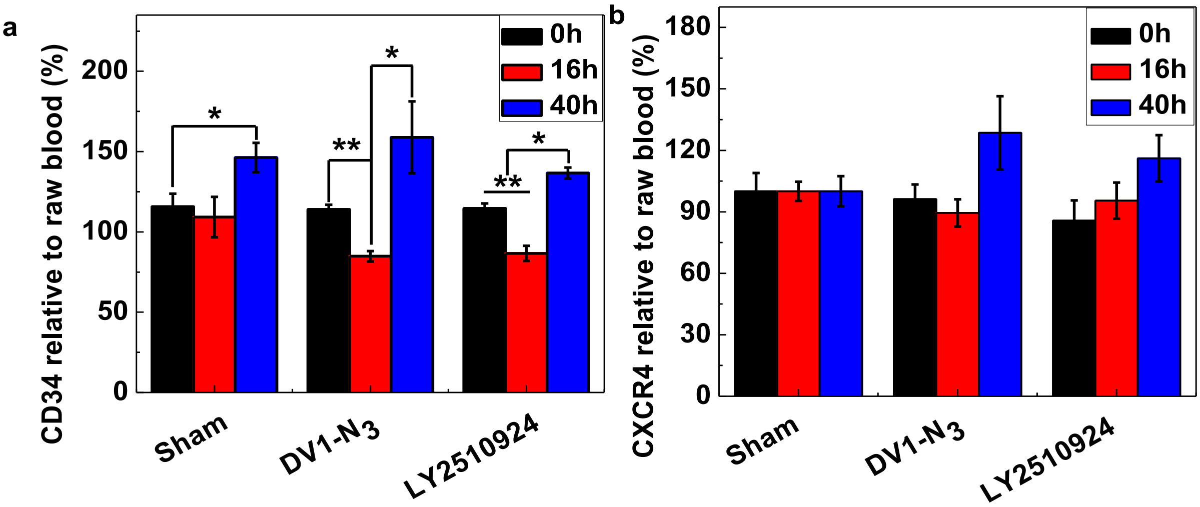

Supplementary Figure 14 CD34^{+} cell migration and CXCR4 expression after DV1-N_3 and LY2510924 administration. (Dosage: 20 mg/kg for DV1-N_3, and 10 mg/kg for LY2510924, which are equivalent to 90μM, the concentration equal to the number of DV1-N_3 on L-DV1-74k (n=6). Error bars represent ±s.e.m. (n≥6, *p<0.05, **p<0.01 by ANOVA with Tukey's post-test).
